# Supplementary material for: 11.4% Efficiency non-fullerene polymer solar cells with trialkylsilyl substituted 2D-conjugated polymer as donor
Source: Nat Commun. 2016 Dec 1;7:13651. doi: 10.1038/ncomms13651 (PMC5146271; doi:10.1038/ncomms13651)
Supplement: Supplementary Information — Supplementary Figures 1-12, Supplementary Tables 1-3, Supplementary References. [file ncomms13651-s1.pdf]

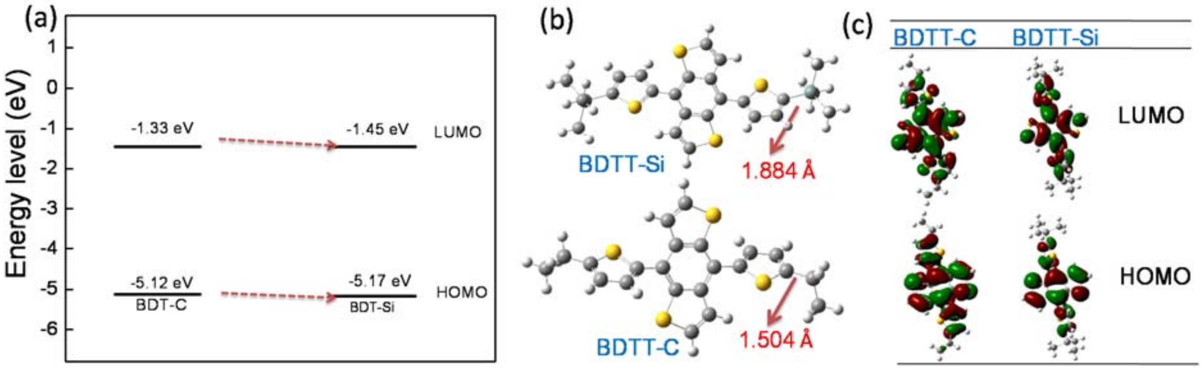

Supplementary Figure 1. (a) HOMO and LUMO energy levels, (b) optimized geometry and (c) frontier molecular orbitals obtained from DFT calculations on BDTT-C and BDTT-Si at B3LYP/6-31G* level with the Gaussian 03 program package. To avoid excessive computation demand, trimethlysilyl group was chosen for BDTT-Si and ethyl group was chosen for BDTT-C.

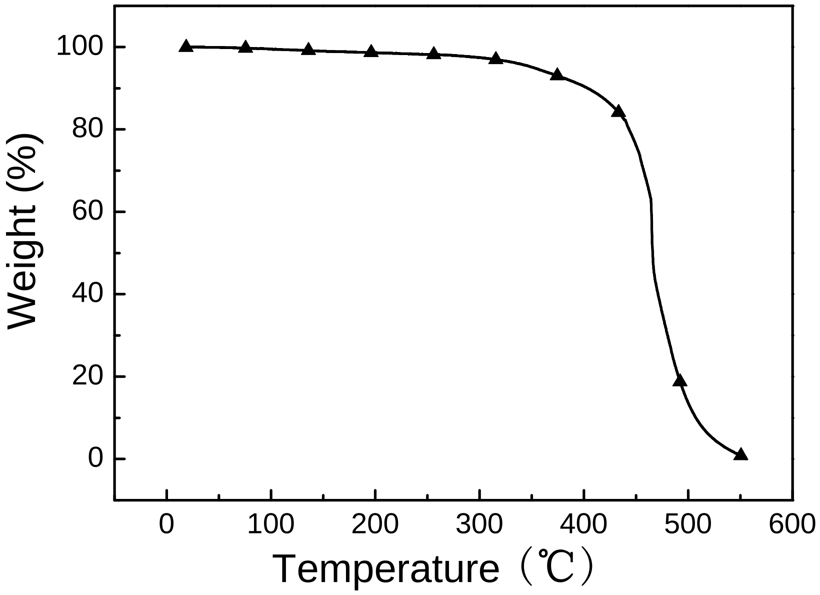

Supplementary Figure 2. TGA plot of the copolymer **J71**

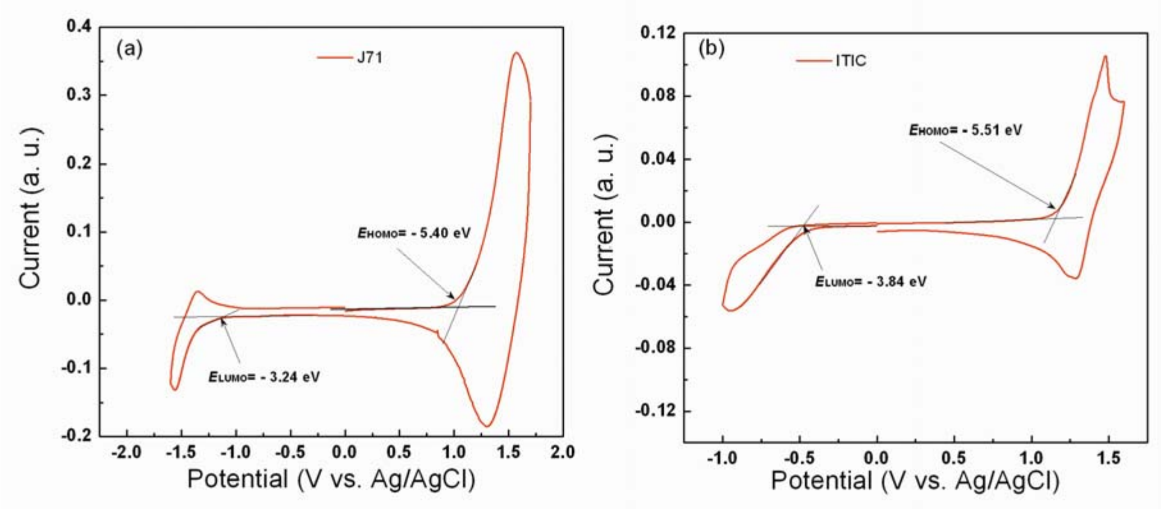

Supplementary Figure 3 Cyclic voltammogram of **J71** (a) and ITIC (b) films on a platinum electrode measured in 0.1 mol L^{-1} Bu_4NPF_6 acetonitrile solutions at a scan rate of 20 mV s^{-1}. The onset potentials were marked in the Figures.

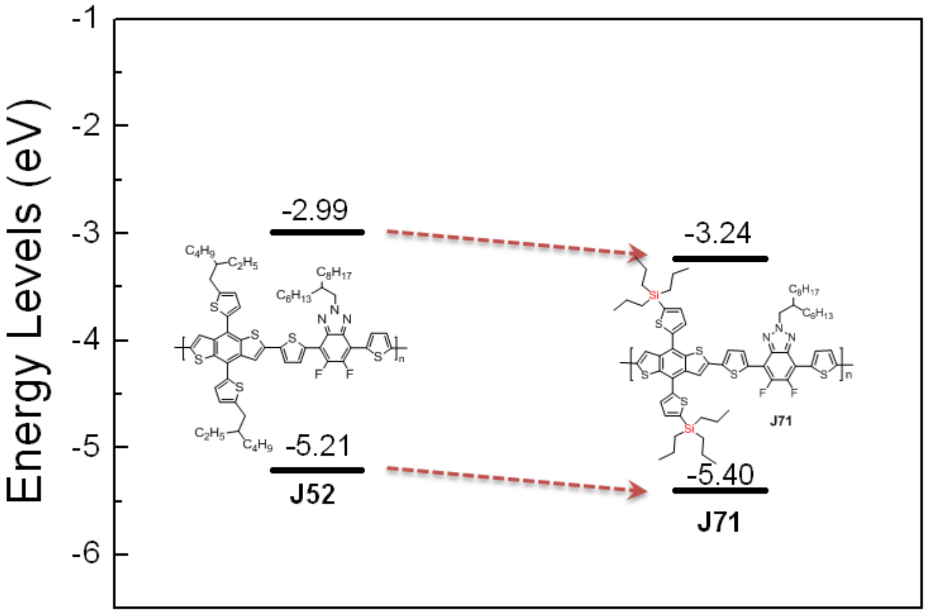

Supplementary Figure 4. Energy level diagrams of **J71** and J52, The insert shows their molecualr structures

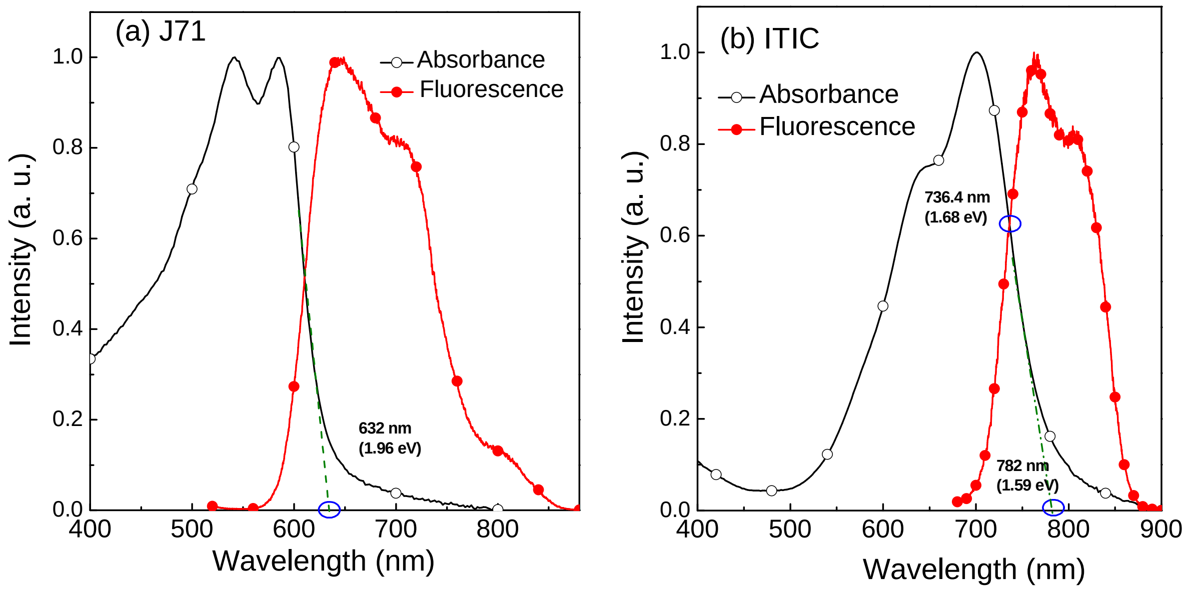

Supplementary Figure 5. UV-visible absorption spectra and fluorescence spectra of (a) **J71** film and (b) ITIC film. The absorption onsets are marked in the Figures.

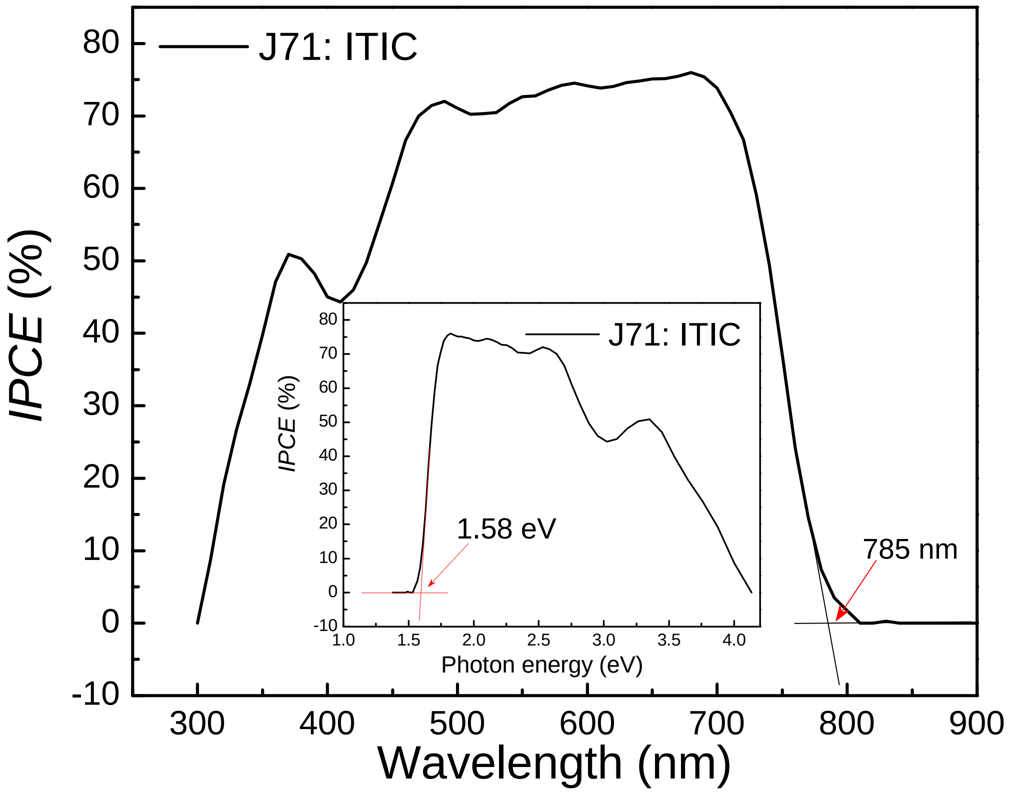

Supplementary Figure 6 IPCE spectrum of the **J71**: ITIC (1:1, w/w), the onset IPCE is marcked as an alternative method to define E_g.

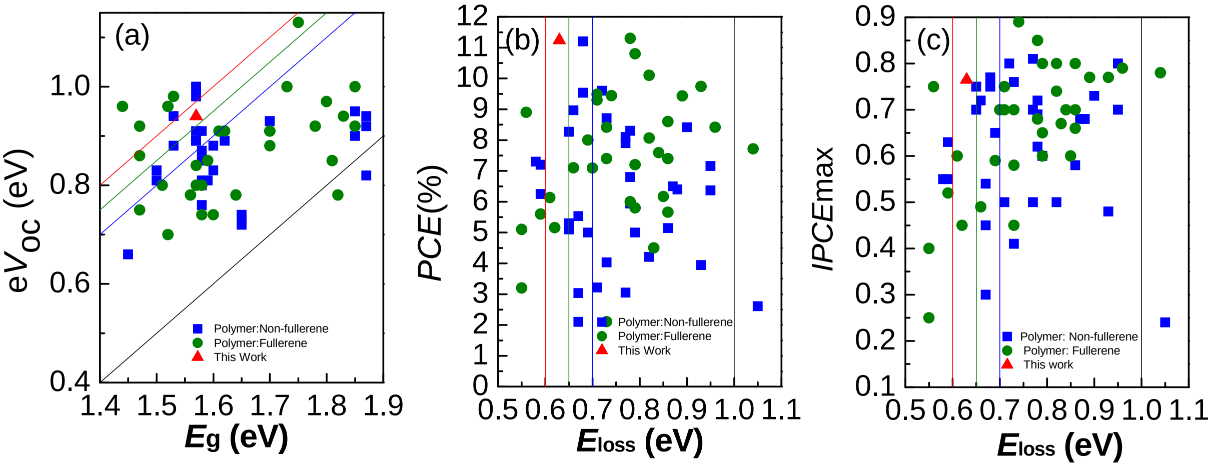

Supplementary Figure 7. Plots of (a) eV_{OC} against E_g, (b) *PCE* and (c) $IPCE_{max}$ against E_{loss} for various PSCs reported in literatures with fullerene or non-fullerene acceptors. The red lines are the lines of E_{loss} = 0.6 eV, the green lines: E_{loss} = 0.65 eV, the blue lines: E_{loss} = 0.70 eV and the black lines: E_{loss} = 1.0 eV.

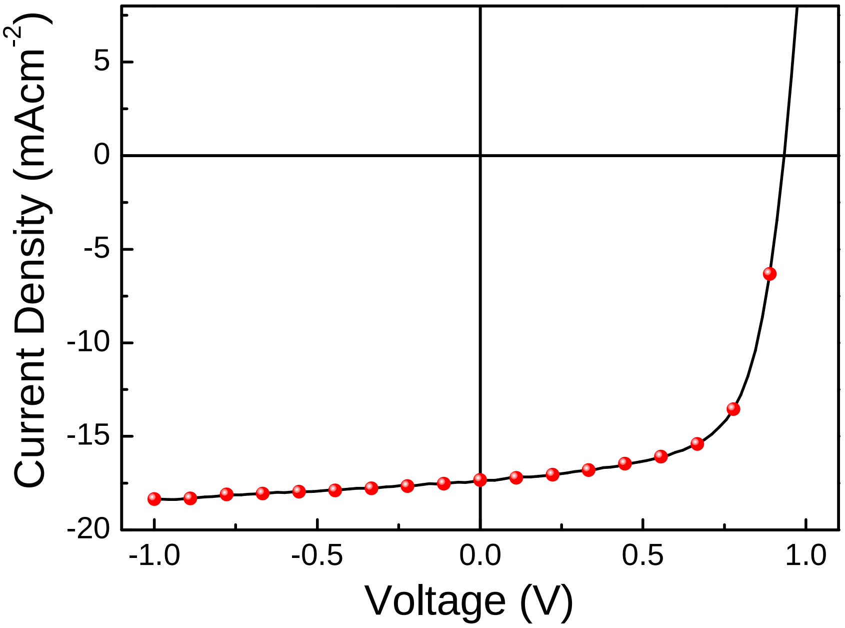

Supplementary Figure 8. *J-V* curve of the inverted structure PSCs of ITO/ZnO /**J71**: ITIC (1:1, w/w) /MoO_3/Al with thermal annealing at 150°C for 10 min, under the illumination of AM 1.5G, 100 mWcm^{-2}. (*PCE* = 10.7% with V_{oc} = 0.93 V, J_{sc} = 17.36 mA cm^{-1}, *FF* = 66.05%)

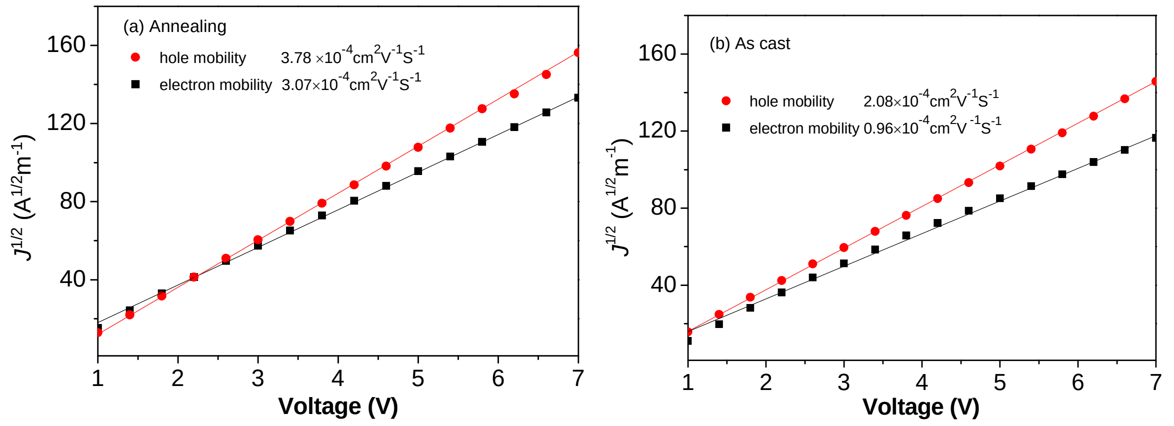

Supplementary Figure 9. $J^{1/2}$~(V_{appl}-V_{bi}-V_s) characteristics for the devices based on the blend films of **J71**:ITIC devices (a) with thermal annealing and (b) without thermal annealing. Solid lines are the fitting lines of the data.

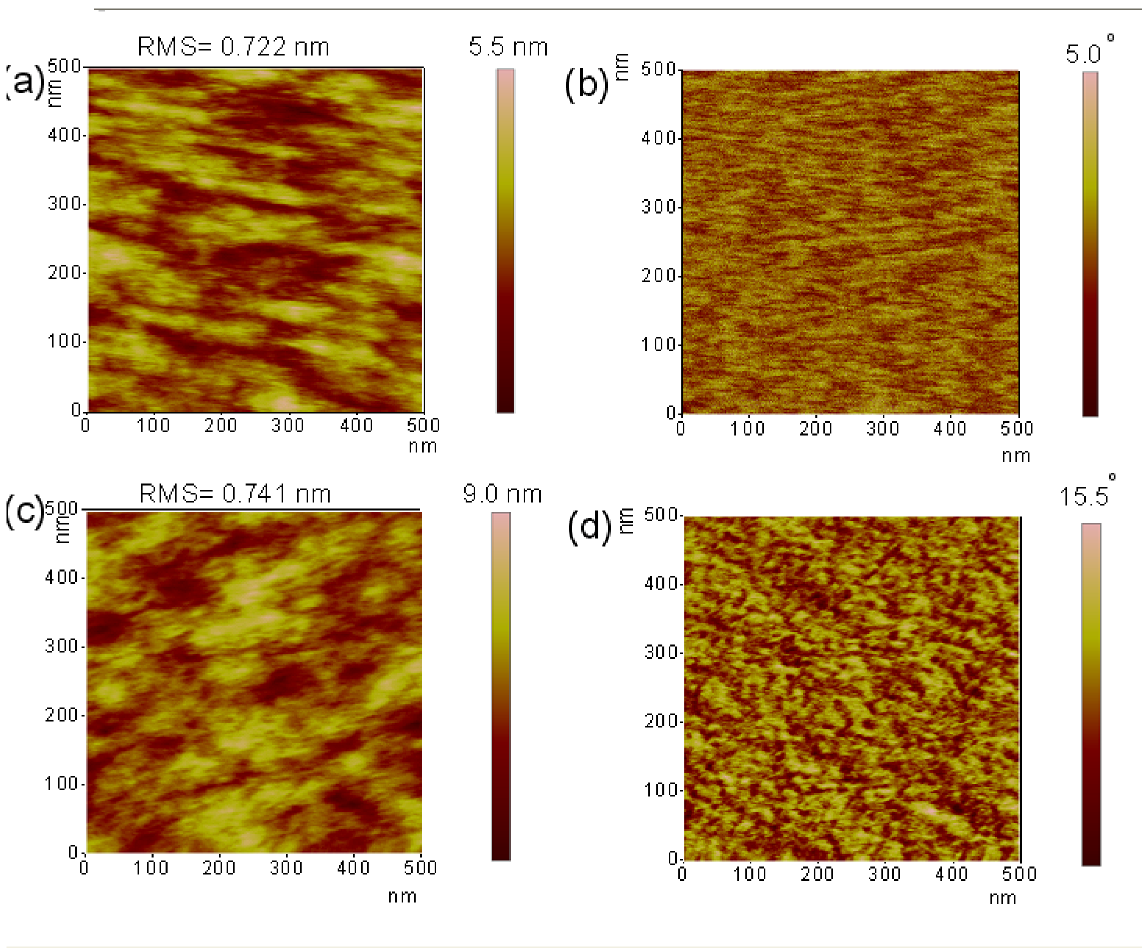

Supplementary Figure 10. Tapping mode AFM images (3×3μm^2) of the **J71**: ITIC blend active layers: topography images of (a) the as cast blend films and (c) thermal annealed films along with (b, d) their corresponding phase images.

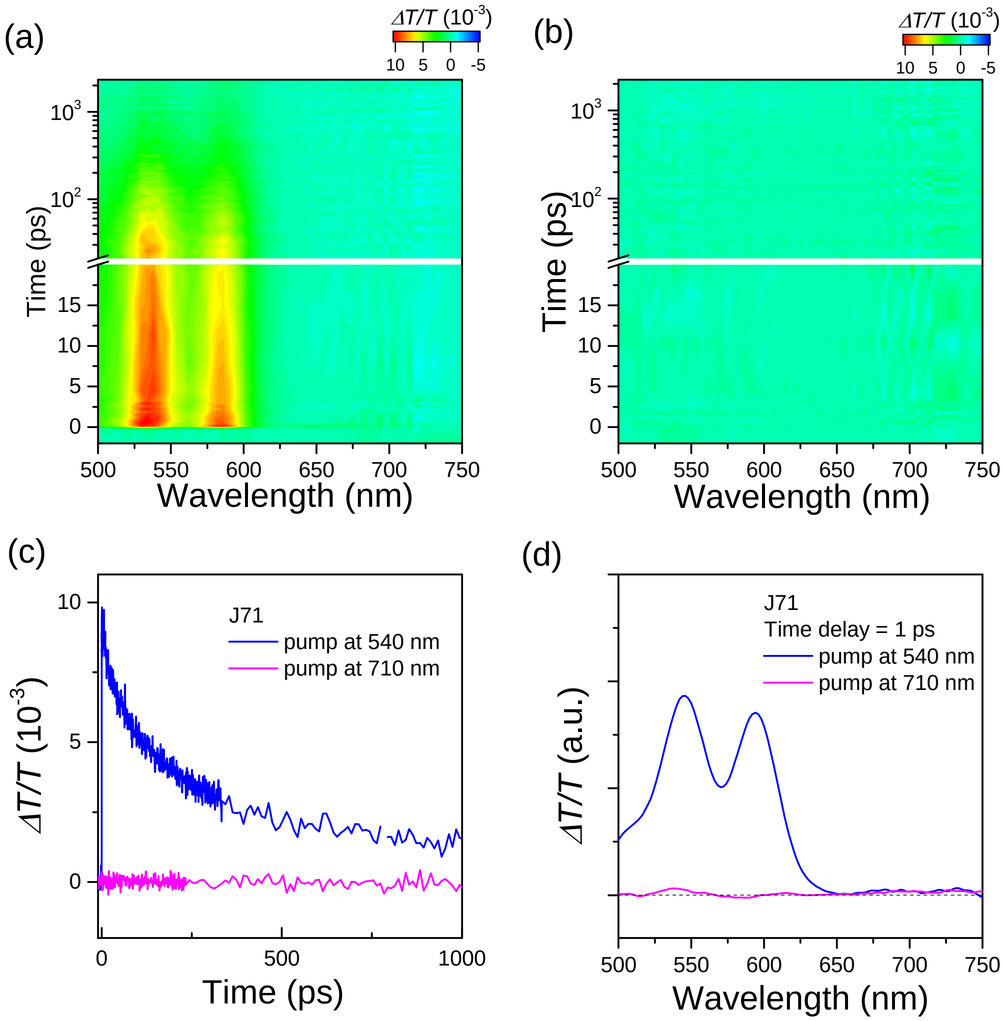

Supplementary Figure 11. TA signal recorded from the near film of **J71** excited by 540 nm (a) and 710 nm (b). (c) Dynamics probed at 540 nm excited by 540 nm and 710 nm. (d) TA spectra at time delay of 1 ps excited by 540 nm and 710 nm.

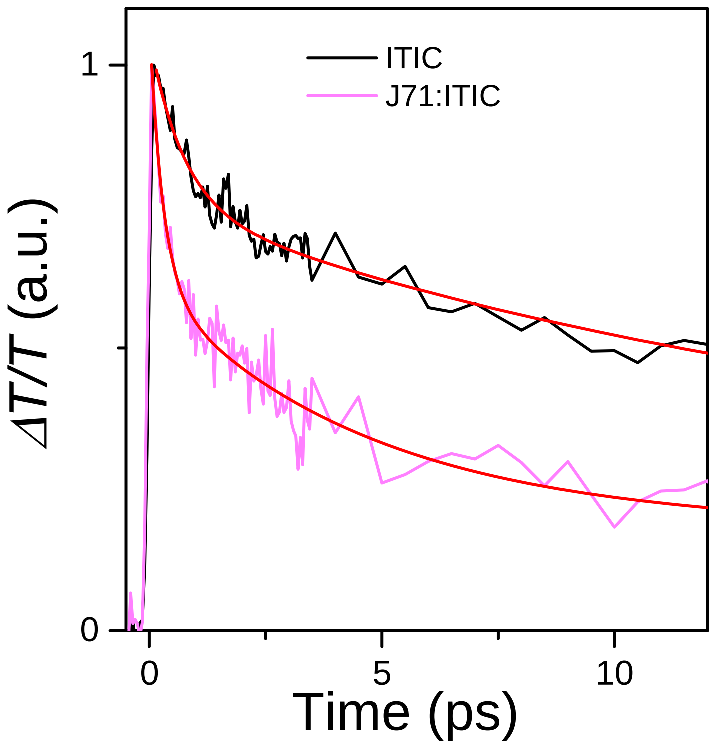

Supplementary Figure 12. The quantify of the early-stage kinetics with a bi-exponetial decay function.(Dynamics probed at 710 nm recorded from the films of neat ITIC and blend **J71**: ITIC (1:1, w/w).)

Supplementary Table 1. Crystal data and structure refinement for BDTT-Si. (CCDC number: 1478875**)**

Identification code	**BDTT-SI**	
Empirical formula	C36 H50 S4 Si2	
Formula weight	667.18	
Temperature	293 K	
Wavelength	0.71073 Å	
Crystal system	Monoclinic	
Space group	C 1 2/c 1	
Unit cell dimensions	*a* = 31.582(17) Å	α= 90°.
	b = 8.491(4) Å	β= 118.635(6)°.
	c = 16.928(9) Å	γ= 90°.
Volume	3984(4) $Å^3$	
Z	4	

Density (calculated)	1.112 Mgm^{-3}
Absorption coefficient	0.321 mm^{-1}
F(000)	1432
Crystal size	0.65 x 0.16 x 0.04 mm^3
Theta range for data collection	2.509 to 25.194°.
Index ranges	-37<=*h*<=36, -10<=*k*<=10, -11<=*l*<=20
Reflections collected	12123
Independent reflections	3569 [*R*(int) = 0.0336]
Completeness to theta = 25.194°	99.4 %
Absorption correction	Semi-empirical from equivalents
Max. and min. transmission	1.000 and 0.595
Refinement method	Full-matrix least-squares on F^2
Data / restraints / parameters	3569 / 199 / 249
Goodness-of-fit on F^2	1.166
Final R indices [I>2sigma(I)]	*R1* = 0.1215, *wR2* = 0.3037
R indices (all data)	*R1* = 0.1337, *wR2* = 0.3141
Extinction coefficient	n/a
Largest diff. peak and hole	0.615 and -0.314 e.$Å^{-3}$

Supplementary Table 2. Atomic coordinates (x 10^4) and equivalent isotropic displacement parameters ($Å^2$x 10^3) for **BDTT-Si**. *U*(eq) is defined as one third of the trace of the orthogonalized ***U**^{ij}* tensor.

	x	*y*	*z*	*U*(eq)
S1	2706(1)	1560(2)	-1555(1)	98(1)
S2	3467(1)	6106(2)	1267(1)	101(1)
Si1	3876(1)	9386(3)	2218(2)	125(1)
C1	3003(2)	3287(8)	-1385(4)	99(2)
C2	3047(2)	4247(6)	-687(3)	70(1)
C3	2774(2)	3439(6)	-274(3)	72(1)
C4	2692(2)	3998(6)	427(3)	68(1)
C5	2418(2)	3052(6)	683(3)	69(1)
C6	2874(2)	5535(6)	867(3)	72(1)
C7	2635(2)	6696(6)	1041(3)	76(1)
C8	2919(2)	8002(7)	1488(3)	80(1)
C9	3387(2)	7895(7)	1676(4)	87(2)
C10	3650(4)	10884(11)	2760(7)	155(4)
C11	3966(4)	12146(13)	3291(9)	186(5)
C12	3744(5)	13172(15)	3716(10)	226(6)
C13	3930(5)	10366(17)	1238(8)	167(5)
C13A	4336(16)	9890(90)	1870(40)	198(9)
C14	4223(8)	11630(30)	1322(13)	226(6)
C14A	4470(20)	10600(110)	1180(40)	230(10)
C15	4332(7)	12020(20)	588(14)	232(7)
C15A	4010(30)	10730(110)	280(40)	254(17)
C16	4477(4)	8460(20)	2811(12)	152(6)
C16A	4400(7)	8450(30)	3223(11)	154(6)
C17	4453(7)	7610(30)	3589(12)	151(6)
C17A	4608(8)	6900(20)	3097(15)	170(6)
C18	4937(9)	6790(40)	4175(17)	215(10)
C18A	5079(9)	6350(40)	3876(19)	213(11)

Supplementary Table 3, *PCE*, V_{OC}, $IPCE_{max}$, E_g (eV) and E_{loss} (eV) for various PSCs

**Active layers**	*PCE* (%)	V_{OC} (V)	$IPCE_{max}$	E_g (eV)	E_{loss} (eV)	References
Bis-PDI-T-EG:PBDTTT-C-T	4.03	0.85	0.41	1.58	0.73	1
PBDTTT-C-T:S(TPA-PDI)	3.22	0.87	0.5	1.58	0.71	2
PBDTT-F-TT:di-PBI	4.21	0.76	0.5	1.58	0.82	3
PTB7-TH:ITIC	6.8	0.81	0.72	1.59	0.78	4
PBDTT-F-TT:TPE-PDI_4	5.53	0.91	0.54	1.58	0.67	5
DBFI-DMT:PSEHTT	6.37	0.92	0.8	1.87	0.95	6
DBFI-S:PSEHTT	2.61	0.82	0.24	1.87	1.05	6
DBFI-MTT:PSEHTT	3.94	0.94	0.48	1.87	0.93	6
PTB7:1	5.14	0.79	0.58	1.65	0.86	7
PBDTT-TT:1	5.94	0.8	0.62	1.58	0.78	7
PffT2-FTAZ-2DT:IEIC	7.3	0.99	0.55	1.57	0.58	8
DBFI-EDOT:PSEHTT	8.1	0.93	0.81	1.7	0.77	6
PDBT-T1:SdiPBI-S	7.16	0.9	0.7	1.85	0.95	9
PDBT-T1:SdiPBI-Se	8.42	0.95	0.73	1.85	0.9	10
PTB7:hPDI3	6.4	0.77	0.68	1.65	0.88	11
PTB7-Th:hPDI3	7.9	0.81	0.7	1.58	0.77	11
PTB7:hPDI4	6.5	0.78	0.68	1.65	0.87	11
PTB7-Th:hPDI4	8.3	0.8	0.69	1.58	0.78	11
J51:N2200	8.27	0.83	0.75	1.48	0.65	12
PBT-3F/$PC_{71}BM$	8.6	0.78	0.7	1.64	0.86	13
PDBT-T1:$PC_{70}BM$	9.74	0.92	0.77	1.85	0.93	14
PBDTTS-TTffBT:$PC_{71}BM$	9.44	0.85	0.89	1.59	0.74	15
PBDT-TS1	9.48	0.8	0.7	1.51	0.71	16
PBDTT-S-TT	8.42	0.84	0.7	1.57	0.73	17
PBDTTT-C-T	7.59	0.74	0.7	1.58	0.84	18
PTB7/$PC_{71}BM$	7.4	0.74	0.66	1.6	0.86	19
PBDTP-DTBT /$PC_{70}BM$	8.07	0.88	0.74	1.7	0.82	20
PBDF-T1:$PC_{70}BM$	9.43	0.94	0.77	1.83	0.89	21
PNOz4T:$PC_{70}BM$	8.9	0.96	0.75	1.52	0.56	22
P3HT:SF(DPPB)4	5.16	1.13	0.45	1.75	0.62	23
PTB7: PiITVT	7.09	0.91	0.7	1.61	0.7	24
PBDT-T8-TPD: PCBM	2.11	1	0.45	1.73	0.73	25
PBDT-T8-TPD: PCBM	6.17	1	0.6	1.85	0.85	25
PBDT-DTNT: $PC_{71}BM$	6	0.8	0.68	1.58	0.78	26
TQ1: $PC_{71}BM$	5.8	0.91	0.6	1.7	0.79	27
PBDTTBT	5.66	0.92	0.8	1.78	0.86	28
PBnDT-DTffBT	7.2	0.91	0.65	1.7	0.79	29
BisDMO-PFDTBT:$PC_{70}B$	4.5	0.97	0.67	1.8	0.83	30

M						
PffBT4T-2OD:$TC_{70}BM$	10.8	0.77	0.8	1.56	0.79	31
PDPP2TzDTP:$TC_{70}BM$	4.9	0.69	0.54	1.28	0.59	31
PB0:ITIC	5.51	0.73	0.6	1.57	0.84	32
J60:ITIC	8.97	0.91	0.72	1.57	0.66	32
J61:ITIC	9.53	0.89	0.77	1.57	0.68	32
DRCN7T:$PC_{61}BM$	9.3	0.91	0.75	1.62	0.71	33
PDPP3TaltTPT:$PC_{70}BM$	8.0	0.75	0.59	1.47	0.69	34
PDPPTPT:$PC_{70}BM$	7.4	0.80	0.58	1.57	0.73	34
PDPP3T:$PC_{70}BM$	7.1	0.67	0.49	1.37	0.66	34
PTB7:DTBT(TDPP)2	3.03	0.83	0.45	1.50	0.67	24
PTB7:DTDfBt T(TDPP)2	5.0	0.81	0.65	1.50	0.69	24
PffT2-FTAZ-2DT:IEIC	7.2	0.98	0.55	1.57	0.68	8
PBDB-T:ITIC	11.2	0.89	0.75	1.57	0.68	35
PCDTBT:NI-T-NI	2.1	1.4	0.3	1.97	0.67	36
PTB7-Th:IDT-IC	3.05	0.83	0.5	1.6	0.77	37
PTB7-Th:IDTIDT-IC	6.25	0.94	0.63	1.53	0.59	37
PCE10:FBM	5.1	0.88	0.70	1.53	0.65	38
PCE10:CBM	5.3	0.88	0.7	1.53	0.65	38
PCE10:CDTBM	5.0	0.66	0.6	1.45	0.79	38
PBDT-T1:IC-C6IDT-IC	8.71	0.89	0.76	1.62	0.73	39
PBDT-T1:ITIC-Th	9.6	0.88	0.8	1.6	0.72	40
PIPCP:$PC_{61}BM$	6.13	0.86	0.6	1.47	0.61	41
PDPP2TzT:$PC_{70}BM$	1.1	0.96	0.05	1.44	0.48	42
PDPP2TzBDT:$PC_{70}BM$	3.2	0.98	0.25	1.53	0.55	42
PDPP2Tz2T:$PC_{70}BM$	5.1	0.92	0.40	1.47	0.55	42
PDPP2TzDTP:$PC_{70}BM$	5.6	0.69	0.52	1.28	0.59	42
PTPD3T:$PC_{71}BM$	7.72	0.78	0.78	1.82	1.04	43
PBTI3T:$PC_{71}BM$	8.84	0.85	0.79	1.81	0.96	43
PNTz4T:$PC_{71}BM$	10.1	0.7	0.8	1.52	0.82	44
PffBT4T-C_9C_{13}:$PC_{71}BM$	11.3	0.77	0.85	1.65	0.88	45
P3TEA: SF-PDI_2	9.5	1.11	0.65	1.72 [a]	0.61	46
PIDTT-TID: $PC_{71}BM$	6.7	1.0	0.70	1.49	0.49	47
J71: ITIC	11.24	0.94	0.76	1.57	0.63	This work

[a] E_g is derived from the crossing point between the absorption and emission spectra.

Supplementary References:

1. Zhang, X., *et al.* A Potential Perylene Diimide Dimer-Based Acceptor Material for Highly Efficient Solution-Processed Non-Fullerene Organic Solar Cells with 4.03% Efficiency. *Adv. Mater.* **25**, 5791-5797 (2013).
2. Lin, Y., *et al.* A Star-Shaped Perylene Diimide Electron Acceptor for High-Performance Organic Solar

Cells. *Adv. Mater.* **26**, 5137-5142 (2014).
3. Zang, Y., *et al.* Integrated Molecular, Interfacial, and Device Engineering towards High-Performance Non-Fullerene Based Organic Solar Cells. *Adv. Mater.* **26**, 5708-5714 (2014).
4. Lin, Y., *et al.* An Electron Acceptor Challenging Fullerenes for Efficient Polymer Solar Cells. *Adv. Mater.* **27**, 1170-1174 (2015).
5. Liu, Y., *et al.* A Tetraphenylethylene Core-Based 3D Structure Small Molecular Acceptor Enabling Efficient Non-Fullerene Organic Solar Cells. *Adv. Mater.* **27**, 1015-1020 (2015).
6. Li, H., *et al.* Fine-Tuning the 3D Structure of Nonfullerene Electron Acceptors Toward High-Performance Polymer Solar Cells. *Adv. Mater.* **27**, 3266-3272 (2015).
7. Zhong, Y., *et al.* Efficient Organic Solar Cells with Helical Perylene Diimide Electron Acceptors. *J. Am. Chem. Soc.* **136**, 15215-15221 (2014).
8. Lin, H., *et al.* High-Performance Non-Fullerene Polymer Solar Cells Based on a Pair of Donor–Acceptor Materials with Complementary Absorption Properties. *Adv. Mater.* **27**, 7299-7304 (2015).
9. Sun, D., *et al.* Non-Fullerene-Acceptor-Based Bulk-Heterojunction Organic Solar Cells with Efficiency over 7%. *J. Am. Chem. Soc.* **137**, 11156-11162 (2015).
10. Meng, D., *et al.* High-Performance Solution-Processed Non-Fullerene Organic Solar Cells Based on Selenophene-Containing Perylene Bisimide Acceptor. *J. Am. Chem. Soc.* **138**, 375-380 (2016).
11. Zhong, Y., *et al.* Molecular helices as electron acceptors in high-performance bulk heterojunction solar cells. *Nature Commun* **6**, 9242 (2015).
12. Gao, L., *et al.* All-Polymer Solar Cells Based on Absorption-Complementary Polymer Donor and Acceptor with High Power Conversion Efficiency of 8.27%. *Adv. Mater.* **28**, 1884–1890 (2016).
13. Zhang, M., Guo, X., Zhang, S. & Hou, J. Synergistic Effect of Fluorination on Molecular Energy Level Modulation in Highly Efficient Photovoltaic Polymers. *Adv. Mater.* **26**, 1118-1123 (2014).
14. Huo, L., *et al.* Single-Junction Organic Solar Cells Based on a Novel Wide-Bandgap Polymer with Efficiency of 9.7%. *Adv. Mater.* **27**, 2938-2944 (2015).
15. Jung, J.W., Liu, F., Russell, T.P. & Jo, W.H. Medium Bandgap Conjugated Polymer for High Performance Polymer Solar Cells Exceeding 9% Power Conversion Efficiency. *Adv. Mater.* **27**, 7462-7468 (2015).
16. Ye, L., Zhang, S., Zhao, W., Yao, H. & Hou, J. Highly Efficient 2D-Conjugated Benzodithiophene-Based Photovoltaic Polymer with Linear Alkylthio Side Chain. *Chem. Mater.* **26**, 3603-3605 (2014).
17. Cui, C., Wong, W.-Y. & Li, Y. Improvement of open-circuit voltage and photovoltaic properties of 2D-conjugated polymers by alkylthio substitution. *Energy Environ. Sci.* **7**, 2276-2284 (2014).
18. Huo, L., *et al.* Replacing Alkoxy Groups with Alkylthienyl Groups: A Feasible Approach To Improve the Properties of Photovoltaic Polymers. *Angew. Chem. Int. Ed.* **50**, 9697-9702 (2011).
19. Liang, Y., *et al.* For the Bright Future—Bulk Heterojunction Polymer Solar Cells with Power Conversion Efficiency of 7.4%. *Adv. Mater.* **22**, E135-E138 (2010).
20. Zhang, M., *et al.* Efficient Polymer Solar Cells Based on Benzothiadiazole and Alkylphenyl Substituted Benzodithiophene with a Power Conversion Efficiency over 8%. *Adv. Mater.* **25**, 4944-4949 (2013).
21. Huo, L., *et al.* Organic Solar Cells Based on a 2D Benzo[1,2-b:4,5-b′]difuran-Conjugated Polymer with High-Power Conversion Efficiency. *Adv. Mater.* **27**, 6969-6975 (2015).
22. Kawashima, K., Tamai, Y., Ohkita, H., Osaka, I. & Takimiya, K. High-efficiency polymer solar cells with small photon energy loss. *Nature Commun.* **6**, 10085 (2015).
23. Li, S., *et al.* A spirobifluorene and diketopyrrolopyrrole moieties based non-fullerene acceptor for

efficient and thermally stable polymer solar cells with high open-circuit voltage. *Energy Environ. Sci.* **9**, 604-610 (2016).

24. Jung, J.W. & Jo, W.H. Low-Bandgap Small Molecules as Non-Fullerene Electron Acceptors Composed of Benzothiadiazole and Diketopyrrolopyrrole for All Organic Solar Cells. *Chem. Mater.* **27**, 6038-6043 (2015).
25. Yuan, J., *et al.* Efficient Polymer Solar Cells with a High Open Circuit Voltage of 1 Volt. *Adv. Funct. Mater.* **23**, 885-892 (2013).
26. Wang, M., *et al.* Donor–Acceptor Conjugated Polymer Based on Naphtho[1,2-c:5,6-c]bis[1,2,5]thiadiazole for High-Performance Polymer Solar Cells. *J. Am. Chem. Soc.* **133**, 9638-9641 (2011).
27. Wang, E., *et al.* An Easily Synthesized Blue Polymer for High-Performance Polymer Solar Cells. *Adv. Mater.* **22**, 5240-5244 (2010).
28. Huo, L., Hou, J., Zhang, S., Chen, H.-Y. & Yang, Y. A Polybenzo[1,2-b:4,5-b']dithiophene Derivative with Deep HOMO Level and Its Application in High-Performance Polymer Solar Cells. *Angew. Chem. Int. Ed.* **49**, 1500-1503 (2010).
29. Zhou, H., *et al.* Development of Fluorinated Benzothiadiazole as a Structural Unit for a Polymer Solar Cell of 7 % Efficiency. *Angew. Chem. Int. Ed.* **50**, 2995-2998 (2011).
30. Chen, M.-H., *et al.* Efficient Polymer Solar Cells with Thin Active Layers Based on Alternating Polyfluorene Copolymer/Fullerene Bulk Heterojunctions. *Adv. Mater.* **21**, 4238-4242 (2009).
31. Liu, Y., *et al.* Aggregation and morphology control enables multiple cases of high-efficiency polymer solar cells. *Nature Commun.* **5**, 5293 (2014).
32. Bin, H., *et al.* Non-Fullerene Polymer Solar Cells Based on Alkylthio and Fluorine Substituted 2D-Conjugated Polymers Reach 9.5% Efficiency. *J. Am. Chem. Soc.* **138**, 4657-4664 (2016).
33. Zhang, Q., *et al.* Small-molecule solar cells with efficiency over 9%. *Nat Photon* **9**, 35-41 (2015).
34. Hendriks, K.H., Heintges, G.H.L., Gevaerts, V.S., Wienk, M.M. & Janssen, R.A.J. High-Molecular-Weight Regular Alternating Diketopyrrolopyrrole-based Terpolymers for Efficient Organic Solar Cells. *Angew. Chem. Int. Ed.* **52**, 8341-8344 (2013).
35. Zhao, W., *et al.* Fullerene-Free Polymer Solar Cells with over 11% Efficiency and Excellent Thermal Stability. *Adv. Mater.* **28**, 4734-4739 (2016).
36. Zhang, X., *et al.* A 1,8-naphthalimide based small molecular acceptor for polymer solar cells with high open circuit voltage. *J. Mater. Chem. C* **3**, 6979-6985 (2015).
37. Li, Y., *et al.* Non-fullerene acceptor with low energy loss and high external quantum efficiency: towards high performance polymer solar cells. *J. Mater. Chem. A* **4**, 5890-5897 (2016).
38. Wang, K., *et al.* π-Bridge-Independent 2-(Benzo[c][1,2,5]thiadiazol-4-ylmethylene) malononitrile Substituted Nonfullerene Acceptors for Efficient Bulk Heterojunction Solar Cells. *Chem. Mater.* **28**, 2200-2208 (2016).
39. Lin, Y., *et al.* A Facile Planar Fused-Ring Electron Acceptor for As-Cast Polymer Solar Cells with 8.71% Efficiency. *J. Am. Chem. Soc.* **138**, 2973-2976 (2016).
40. Lin, Y., *et al.* High-Performance Electron Acceptor with Thienyl Side Chains for Organic Photovoltaics. *J. Am. Chem. Soc.* **138** 4955-4961 (2016).
41. Wang, M., *et al.* High Open Circuit Voltage in Regioregular Narrow Band Gap Polymer Solar Cells. *J. Am. Chem. Soc.* **136**, 12576-12579 (2014).
42. Li, W., Hendriks, K.H., Furlan, A., Wienk, M.M. & Janssen, R.A.J. High Quantum Efficiencies in Polymer Solar Cells at Energy Losses below 0.6 eV. *J. Am. Chem. Soc.* **137**, 2231-2234 (2015).
43. Guo, X., *et al.* Polymer solar cells with enhanced fill factors. *Nature Photon* **7**, 825-833 (2013).

44. Vohra, V., *et al.* Efficient inverted polymer solar cells employing favourable molecular orientation. *Nature Photon.* **9**, 403-408 (2015).
45. Zhao, J., *et al.* Efficient organic solar cells processed from hydrocarbon solvents. *Nature Energy* **1**, 15027 (2016).
46. Liu, J., *et al.* Fast charge separation in a non-fullerene organic solar cell with a small driving force. *Nature Energy* **1**, 16089 (2016).
47. Wang, C., *et al.* Low Band Gap Polymer Solar Cells With Minimal Voltage Losses. *Adv. Energ. Mater.*, 6, 1600148 (2016).
